# Retraction: Antimicrobial activities of different solvent extracts from stem and seeds of *Peganum Harmala* L

**DOI:** 10.1371/journal.pone.0273538

**Published:** 2022-08-31

**Authors:** The PLOS ONE Editors

The *PLOS ONE* Editors retract this article [1] because it was identified as one of a series of submissions for which we have concerns about authorship, competing interests, and peer review. We regret that the issues were not addressed prior to the article’s publication.

AFN and SF did not agree with the retraction. MArif and XW responded but expressed neither agreement nor disagreement with the editorial decision. MSKK, SUK, SSaeed, AMK, RAK, MAfzal, MAZ, HOE, SShokralla, AA, and AG either did not respond directly or could not be reached.
